# Identifying platelet-derived factors as amplifiers of *B. burgdorferi*-induced cytokine production

**DOI:** 10.1093/cei/uxac073

**Published:** 2022-08-24

**Authors:** Mariska Kerstholt, Freek R van de Schoor, Marije Oosting, Simone J C F M Moorlag, Yang Li, Martin Jaeger, Wouter A van der Heijden, Rahajeng N Tunjungputri, Jéssica C dos Santos, Brenda Kischkel, Hedwig D Vrijmoeth, M E Baarsma, Bart-Jan Kullberg, Mihaela Lupse, Joppe W Hovius, Cees C van den Wijngaard, Mihai G Netea, Quirijn de Mast, Leo A B Joosten

**Affiliations:** Department of Internal Medicine and Radboudumc Center for Infectious diseases (RCI), Radboud University Medical Center, Nijmegen, The Netherlands; Department of Internal Medicine and Radboudumc Center for Infectious diseases (RCI), Radboud University Medical Center, Nijmegen, The Netherlands; Department of Internal Medicine and Radboudumc Center for Infectious diseases (RCI), Radboud University Medical Center, Nijmegen, The Netherlands; Department of Internal Medicine and Radboudumc Center for Infectious diseases (RCI), Radboud University Medical Center, Nijmegen, The Netherlands; Department of Internal Medicine and Radboudumc Center for Infectious diseases (RCI), Radboud University Medical Center, Nijmegen, The Netherlands; Department of Computational Biology for Individualised Medicine, Centre for Individualised Infection Medicine (CiiM) and TWINCORE, Joint Ventures Between the Helmholtz-Centre for Infection Research (HZI) and the Hannover Medical School (MHH), Hannover, Germany; Department of Internal Medicine and Radboudumc Center for Infectious diseases (RCI), Radboud University Medical Center, Nijmegen, The Netherlands; Department of Internal Medicine and Radboudumc Center for Infectious diseases (RCI), Radboud University Medical Center, Nijmegen, The Netherlands; Department of Internal Medicine and Radboudumc Center for Infectious diseases (RCI), Radboud University Medical Center, Nijmegen, The Netherlands; Center for Tropical and Infectious Diseases (CENTRID), Faculty of Medicine Diponegoro University, Dr. Kariadi Hospital, Semarang, Indonesia; Department of Internal Medicine and Radboudumc Center for Infectious diseases (RCI), Radboud University Medical Center, Nijmegen, The Netherlands; Department of Internal Medicine and Radboud Institute of Molecular Life Sciences (RIMLS), Radboud University Medical Centre, Nijmegen, The Netherlands; Department of Internal Medicine and Radboudumc Center for Infectious diseases (RCI), Radboud University Medical Center, Nijmegen, The Netherlands; Amsterdam Institute of Infection and Immunology, Center for Experimental and Molecular Medicine, Amsterdam UMC, University of Amsterdam, Amsterdam, The Netherlands; Department of Internal Medicine and Radboudumc Center for Infectious diseases (RCI), Radboud University Medical Center, Nijmegen, The Netherlands; Department of Infectious Diseases, University of Medicine and Pharmacy ‘Iuliu Hatieganu’, Cluj-Napoca, Romania; Amsterdam Institute of Infection and Immunology, Center for Experimental and Molecular Medicine, Amsterdam UMC, University of Amsterdam, Amsterdam, The Netherlands; National Institute for Public Health and the Environment (RIVM), Center of Infectious Disease Control, Bilthoven, The Netherlands; Department of Internal Medicine and Radboudumc Center for Infectious diseases (RCI), Radboud University Medical Center, Nijmegen, The Netherlands; Department for Immunology and Metabolism, Life and Medical Sciences Institute (LIMES), University of Bonn, Germany; Department of Internal Medicine and Radboudumc Center for Infectious diseases (RCI), Radboud University Medical Center, Nijmegen, The Netherlands; Department of Internal Medicine and Radboudumc Center for Infectious diseases (RCI), Radboud University Medical Center, Nijmegen, The Netherlands

**Keywords:** *Borrelia burgdorferi*, Lyme disease, metabolism, trained immunity, platelets, CXCL7

## Abstract

Previous studies have shown that monocytes can be ‘*trained*’ or tolerized by certain stimuli to respond stronger or weaker to a secondary stimulation. Rewiring of glucose metabolism was found to be important in inducing this phenotype. As we previously found that *Borrelia burgdorferi (B. burgdorferi*), the causative agent of Lyme borreliosis (LB), alters glucose metabolism in monocytes, we hypothesized that this may also induce long-term changes in innate immune responses. We found that exposure to *B. burgdorferi* decreased cytokine production in response to the TLR4-ligand lipopolysaccharide (LPS). In addition, *B. burgdorferi* exposure decreased baseline levels of glycolysis, as assessed by lactate production. Using GWAS analysis, we identified a gene, microfibril-associated protein 3-like (MFAP3L) as a factor influencing lactate production after *B. burgdorferi* exposure. Validation experiments proved that MFAP3L affects lactate- and cytokine production following *B. burgdorferi* stimulation. This is mediated by functions of MFAP3L, which includes activating ERK2 and through activation of platelet degranulation. Moreover, we showed that platelets and platelet-derived factors play important roles in *B. burgdorferi-*induced cytokine production. Certain platelet-derived factors, such chemokine C-X-C motif ligand 7 (CXCL7) and (C-C motif) ligand 5 (CCL5), were elevated in the circulation of LB patients in comparison to healthy individuals.

## Teaser

In this study, we observed that *B. burgdorferi*decreased cytokine production in response to the TLR4-ligand lipopolysaccharide (LPS) and glycolysis was lowered by cells exposed to *B. burgdorferi*compared to medium control. We found a gene, microfibril-associated protein 3-like (MFAP3L), that influences lactate production following *B. burgdorferi*stimulation and MFAP3L could mediate this effect by activating ERK2 and platelet activation and degranulation. A specific platelet-derived factor, CXCL7, was associated with *B. burgdorferi*-induced cytokine production and was found to be elevated in plasma and serum of erythema migrans patients, the most common disease manifestation of Lyme borreliosis."

## Graphical Abstract

**Graphical Abstract:**
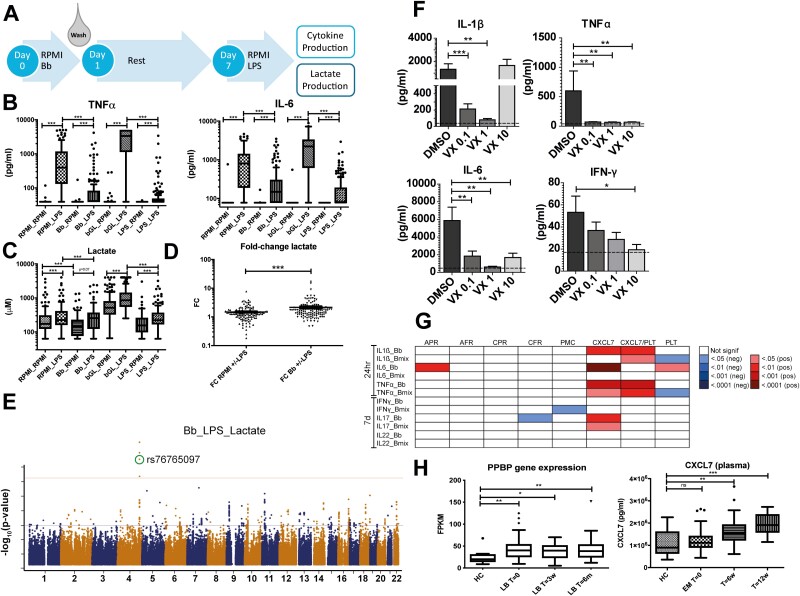

## Introduction

*Borrelia burgdorferi* is the causative agent of Lyme borreliosis (LB), the most common vector-borne disease in the Northern Hemisphere [1, 2]. LB often presents with a centrifugally expanding skin rash called erythema migrans (EM). The infection can develop into more severe manifestations such as acrodermatitis chronica atrophicans, Lyme arthritis, Lyme carditis, or neuroborreliosis [3]. Generally, antibiotic treatment for LB is effective, yet a small percentage of patients experience persisting symptoms even after treatment [4, 5].

Since *B. burgdorferi* is not known to produce toxic factors [6–8], it is likely that the majority of LB symptoms can be attributed to the host response against the pathogen [9–11]. In addition, most evidence indicates that antibiotic-refractory Lyme arthritis (ARLA) is caused by an aberrant inflammatory response rather than persistent infection [12, 13]. Some studies suggest that immune responses also are involved in Post-Treatment Lyme Disease Syndrome (PTLDS) [14, 15]. The host immune response, therefore, plays a crucial role in both the initiation and pathology of LB.

Metabolic profiling of white blood cells has demonstrated the important role of cellular metabolism for immune cell activation. We recently found alterations in glucose and glutathione metabolism in primary monocytes after stimulation with *B. burgdorferi*. These metabolic routes were proved to be essential for the inflammatory response [16] and were identified *in vivo* in the circulation of patients with EM [17]. Recently, studies showed that a certain metabolic profile could distinguish healthy controls from EM patients and a different tick-transmitted disease, southern tick-associated rash illness (STARI) [18].

Importantly, the metabolism of glucose was also shown to play a major role in the induction of trained immunity, the long-term functional reprogramming of innate immune cells conferred by infection or vaccination [19, 20]. We therefore hypothesized that *B. burgdorferi*-induced changes in cellular metabolism might also alter monocyte responses to a secondary stimulus. To investigate this, we studied a cohort of healthy volunteers from whom primary monocytes were exposed to *B. burgdorferi* followed by secondary stimulation with *Escherichia coli* lipopolysaccharide (LPS). After a resting period, the cells were restimulated with an unrelated stimulus (in this case the TLR4 ligand LPS) and the production of cytokines and lactate were analyzed. Next, we performed a genome-wide association study (GWAS) to determine the genetic factors that play a role in these responses. The genetic factors that we identified were then validated in additional *in vitro* experiments.

In this study, we aim to show the response of primary monocytes primed by *B. burgdorferi* followed by a secondary stimulus investigating the role of *B. burgdorferi* in trained innate memory.

## Materials and Methods

### Study population

The 200 Functional Genomics (200FG) cohort consists of healthy volunteers of Western European ancestry, working as foresters across the Netherlands. As their occupation results in a higher risk of getting exposed to *B. burgdorferi*, *s*erology was performed on all subjects in this cohort to determine previous exposure to the spirochete. None of the volunteers included had signs or symptoms of active *B. burgdorferi* infection at the moment of sample collection.

500FG is a cohort of 500 healthy individuals of Western-European ancestry from the Human Functional Genomics Project (www.humanfunctionalgenomics.org). General characteristics of this cohort have been described previously [21].

LB patient material (heparin plasma) was available from 94 adult patients with physician-confirmed EM included in the LymeProspect study [22]. Blood was drawn at the time of diagnosis, preferably before but ultimately within seven days after the beginning of antibiotic therapy. Follow-up blood samples were acquired six and twelve weeks after the first phlebotomy. For comparison, healthy controls from the Netherlands were used, who were included in the VICTORY study [23]. Additionally, healthy controls from the laboratory were used as a control group.

In addition, serum samples were available from a cohort of Romanian patients with clinically confirmed EM. Baseline samples were obtained before start of antibiotic treatment, and follow-up samples at 2, 6, and 12 weeks from baseline. Serum samples from tick-bitten healthy controls were collected at one time point.

### Culture of *B. burgdorferi* spirochetes

*B. burgdorferi*, ATCC strain 35210 (ATCC, Wesel, Germany) was cultured at 24°C in Barbour-Stoenner-Kelley (BSK)-H medium (Sigma-Aldrich) supplemented with 6% rabbit serum until spirochete growth commenced. Cells were then grown at 34°C to late logarithmic phase, at which point the spirochetes were checked for motility by dark-field microscopy and harvested. Spirochetes were quantified using a Petroff-Hauser counting chamber, washed three times with PBS and stored at -80°C.

### Isolation of PBMCs and monocytes

Peripheral blood mononuclear cells (PBMCs) were isolated from fresh blood from healthy volunteers (cohort subjects) or buffy coats (for validation experiments). Blood was diluted with sterile PBS (1:1) and a density centrifugation was applied over Ficoll-Paque (Pharmacia Biotech) as described previously [24]. Next, the interphase containing the PBMCs was collected, washed with ice-cold PBS and cells were resuspended in RPMI 1640 medium supplemented with gentamycin 10 mg/mL, L-glutamine 10 mM and pyruvate 10 mM. Cells were counted in a Coulter counter (Coulter Electronics) and adjusted to a concentration of 5 × 10^6^ cells/mL and seeded in round-bottom 96-well plates (5 × 10^5^ cells/well).

For the experiments using platelet-poor PBMCs, PBMCs were spun down at low speed (156 × *g*, without brake) for 15 min to pellet the PBMCs but not the platelets. Cells were washed once with PBS, then resuspended in RPMI, and treated as described before.

The protocol showing how to train human monocytes has been published recently [25]. For experiments using monocytes, PBMCs were seeded in flat-bottom plates and left to adhere for one hour at 37°C. Subsequently, wells were washed three times with 200 µL warm PBS to remove non-adherent cells and increase purity of the monocyte fraction. After one hour, cells were stimulated with RPMI (negative control) or *B. burgdorferi* (10^6^ sp/mL, MOI = 1), β-glucan (bGL, 2 µL) or 10 ng/ml LPS (lipopolysaccharide, *E. coli* serotype 055:B5, Sigma) in medium supplemented with 10% pooled human serum (HS). Plates were then incubated for 24 hours at 37°C, after which the stimuli were washed away with warm PBS and fresh medium containing 10% HS was added. Cells were maintained at 37°C in the incubator for a total of six days, with one medium refreshment at day three. After the resting period, cells were restimulated for 24 hours with 10 ng/mL LPS. Finally, after restimulation, supernatants were collected and stored at −20°C until assayed.

### Reagents

In intervention experiments, cells were pretreated for 1 hour with VTX-11e (TCS ERK 11e, Tocris Bioscience), rabbit polyclonal anti-MFAP3L antibody (Abcam), recombinant human Chemokine C-X-C motif ligand 7 (CXCL7) (R&D systems), mouse monoclonal anti-CXCL7 (clone # 59418, R&D systems) or appropriate control (DMSO vehicle control and IgG control respectively) prior to the initial stimulation with *B. burgdorferi* or RPMI.

### Cytokine measurements

Cytokine concentrations in cell culture supernatants were measured by sandwich ELISA using commercial kits specific for IL-6, IFN-γ (Sanquin, Amsterdam, the Netherlands), IL-1β, TNFα or CXCL7 (R&D Systems, Abingdon, United Kingdom) according to the manufacturers’ instructions. Absorbance was measured using an Infinite^®^ 200 PRO microplate reader (Tecan, Giessen, the Netherlands). CXCL7 and CCL5 were measured in heparin plasma samples by Luminex Assay (R&D Systems, Abingdon, United Kingdom). The overnight shipment time of these plasma samples was matched to the pre-centrifugation time of the control plasma samples.

### Lactate measurements

Lactate concentrations in cell culture supernatants were quantified using Amplex^®^ Red reagent (0.2 mM, Thermo Fisher Scientific). First, lactate oxidase (2 U/mL, derived from *Pediococcus* sp., Sigma) was used to break down lactate, yielding hydrogen peroxide. In the presence of horseradish peroxidase (0.2 U/mL HRP, Thermo Fisher Scientific), hydrogen peroxide reacts with Amplex^®^ Red to generate the fluorescent product resorufin. Because the oxidase- and peroxidase-mediated reactions are coupled, the amount of fluorescence directly correlates to the amount of lactate. Cell-free medium samples, incubated for the same amount of time, were included to allow for background correction. Fluorescence was measured (Ex: 570 nM, Em: 585 nM) and concentrations were derived from a standard curve of sodium-L-lactate (Sigma).

### Genotyping, imputation and QTL mapping

Individuals of 200FG cohort were genotyped using the Illumina HumanOmniExpressExome-8 v1.0 and the data was imputed, as previously described [26]. Raw cytokine and lactate levels were first log-transformed. Then, the ratio of cytokine/lactate measurements between the second and the first stimulation was computed to capture the induction of tolerance. The ratio of log-transformed cytokine/lactate data was mapped to genotype data using a linear model with age and gender as covariates. The genome-wide significance was based on a threshold of *P* value <5 × 10^−8^.

### RNA sequencing and eQTL expression analysis

The RNA sequencing dataset and eQTL mapping were described previously [27]. Briefly, The sequencing reads were mapped to human reference genome NCBI build 37 using STAR v2.3.1 [28]. Gene expression was estimated using HTSeq count [29] using Ensembl GRCh37.71 gene annotation. Gene expression data was TMM (trimmed mean of M values) normalized and log2-transformed. Subsequently, eQTL mapping was done by using linear regression model with age and gender as covariates. Gene function was predicted using a previously described gene network prediction model based on pathways and gene sets from the Gene Ontology (GO), Kyoto Encyclopedia of Genes and Genomes (KEGG), Reactome and Biocarta databases [30]. eQTL threshold is defined as *P*<-1 × 10^4^.

### Western blot

Stimulated PBMCs (5x10^6^) were lysed in 100 µL lysis buffer (50 mM Tris, 150 mM NaCl, 2 mM EDTA, 2 mM EGTA, 10% glycerol, 1% Triton X-100, 40 mM α-glycerophosphate, 50 mM sodium fluoride, 200 μM sodium vanadate, 10 μg/mL leupeptin, 423 10 μg/mL aprotinin, 1 μM pepstatin A, and 1 mM phenylmethylsulfonyl fluoride) and stored at -80°C until further use. Lysates were centrifuged (10 min, 14000 rpm, 4°C) to eliminate cell debris. Supernatans were used for Western blot analysis. Bio-Rad 4-15% polyacrylamide gels were used to load equal amounts of protein. Next, the separated proteins were transferred to Nitrocellulose (Bio-Rad) membranes and the membrane was blocked for 1 hour with 5% BSA (bovine serum albumin, Sigma) in TBS-Tween buffer (TBS-T). This was incubated overnight with a primary antibody (RSK1 (D6D5) and Phospho-p90RSK (Thr359) (D1E9), both Cell Signaling or rabbit anti-actin, Sigma) at a dilution of 1:1000 in blocking buffer (TBS-T with 5% BSA). Blots were washed following overnight incubation in TBS-T three times and incubated with HRP-conjugated anti-rabbit antibody (1:5000; Sigma) in 5% milk in TBS-T for 1 hour. Blots were developed with ECL (Bio-Rad) according to the manufacturer’s instructions and results were quantified with Image Lab (version 5.2.1).

### Measurement of platelet function and activation in 500FG subjects

Venous blood was collected in citrated Vacutainer tubes (3.2% sodium citrate, Becton Dickinson, USA). Platelet reactivity was determined by a whole blood flow cytometry assay that has been described earlier [31]. In short, platelet membrane expression of the alpha-granule protein P-selectin and the binding of fibrinogen to the activated integrin αIIbβ3 are measured in unstimulated samples and after *ex vivo* platelet stimulation by eight increasing concentrations of adenosine diphosphate (ADP, 7.8-125 µM, Sigma) or crosslinked collagen-related peptide (CRP-XL, 9-625ng/mL). Whole blood was added to a mixture of HEPES-buffered saline and saturating concentrations of PE-labeled anti-CD62P (P-selectin; Bio-Legend, San Diego, USA), FITC-labeled anti-fibrinogen (DAKO Ltd., High Wycombe, UK) and PC7-labeled anti-CD61 (platelet identification marker; Beckman Coulter, France). After 20 minutes incubation at room temperature, 0.2% paraformaldehyde was added, and samples were analyzed using a FC500 flow cytometer (Beckman Coulter) within 4 hours. Platelets were gated based on their forward- and sideward-scatter properties and positivity for CD61, which was defined as a median fluorescence intensity (MFI) exceeding that of the matched isotype control; 10.000 platelets were measured in each sample. Next, ADP- and CRP-XL-induced platelet P-selectin (termed as APR and CPR) and platelet-fibrinogen reactivity to either ADP or CRP-XL (termed as AFR and CFR) were determined by calculating the area under the curve (AUC) from the MFI of CD62P or fibrinogen on CD61-positive events generated from the increasing concentrations of ADP or CRP-XL.

The formation of platelet mediator concentrate (PMC), which is considered a sensitive marker for platelet activation, was determined by incubating citrated whole blood with PC7-labelled anti-CD61 and PE-labelled anti-CD14 (a glycosylphosphatidylinositol (GPI)-linked membrane glycoprotein; Bio-Legend) as a monocyte identification marker. Optilyse B (Beckman Coulter) was added after 30 min followed by distilled water. The PMC formation was quantified based on the MFI of CD61 on CD14-positive cells.

Plasma concentrations of the platelet α-granule CXCL7, a plasma soluble marker for platelet activation, and thrombin-antithrombin (TAT) complexes, a marker for coagulation activation, from all samples were measured using ELISA as previously described [32]. Anti-human β-thromboglobulin (MAB393, BAF393) was purchased from R&D systems, Abingdon, UK, and sheep anti-human thrombin antibodies was purchased from Kordia/Affinity Biologicals (Ancaster, Ontario, Canada).

### Platelet stimulation and activation assay for validation experiments

Venous blood was collected in citrated Vacutainer tubes (3.2% sodium citrate, Becton Dickinson, USA). The tubes were spun down at low speed (156xg) for 15 minutes at room temperature (RT) with no brake to obtain the platelet-rich plasma (PRP). Platelet concentration and leukocyte contamination were measured with a Sysmex XN450 hematology analyzer. Platelet concentration was adjusted to 2 × 10^5^ plt/µL using platelet-poor plasma, obtained after centrifuging the remaining blood for 10 min at 2000xg, without break. After isolation, platelets were incubated for 3 hours with increasing doses of *B. burgdorferi* and adenosine-di-phosphate (ADP) as a positive control. After 3 hours, samples were collected for flow cytometry analysis and the remainder was spun down to collect cell-free supernatants.

Platelet activation was determined by the membrane expression of the alpha-granule protein P-selectin and platelet-fibrinogen binding. In short, isolated platelets were incubated with an antibody cocktail consisting of PE-labeled anti-CD62P (Bio-Legend, San Diego, USA), FITC-labeled anti-fibrinogen (Dako, Glostrup, Denmark) and PC7-labeled anti-CD61. After incubation for 20min at RT, cells were fixed with 0.2% paraformaldehyde and analyzed using a FC500 flow cytometer (Beckman Coulter, France). Platelets were gated based on their forward- and sideward scatter (FSC/SSC) properties and positivity for the platelet-marker CD61.

### Cytotoxicity assay

Cell survival was analyzed using the CytoTox 96^®^ Non-Radioactive Cytotoxicity Assay (Promega, Leiden, the Netherlands) according to the manufacturer’s instruction. This assay is based on the measurement of lactate dehydrogenase (LDH) in cell-free supernatant. LDH is a stable cytosolic enzyme and is only released into the supernatant after cell lysis and can therefore be used as a measure of cell death.

### Statistical analysis

Statistical analysis was performed using GraphPad Prism (version 5.03, San Diego, California, USA). QTL mapping was performed with statistical programming language R (R Core Team, 2012). Bivariate correlations were tested using Spearman’s rho correlation coefficient. Comparison between groups was done by Mann-Whitney U test or Student’s T-test depending on data distribution. Paired T-test or Wilcoxon matched-pairs signed rank test were used to compare continuous parameters from the same individual with different stimulations again depending on data distribution. Data are expressed as mean ± SEM, unless stated otherwise. P-values less than 0.05 were considered statistically significant.

## Results

### *B. burgdorferi* exposure decreases cytokine responses to a secondary stimulus

**Figure 1:**
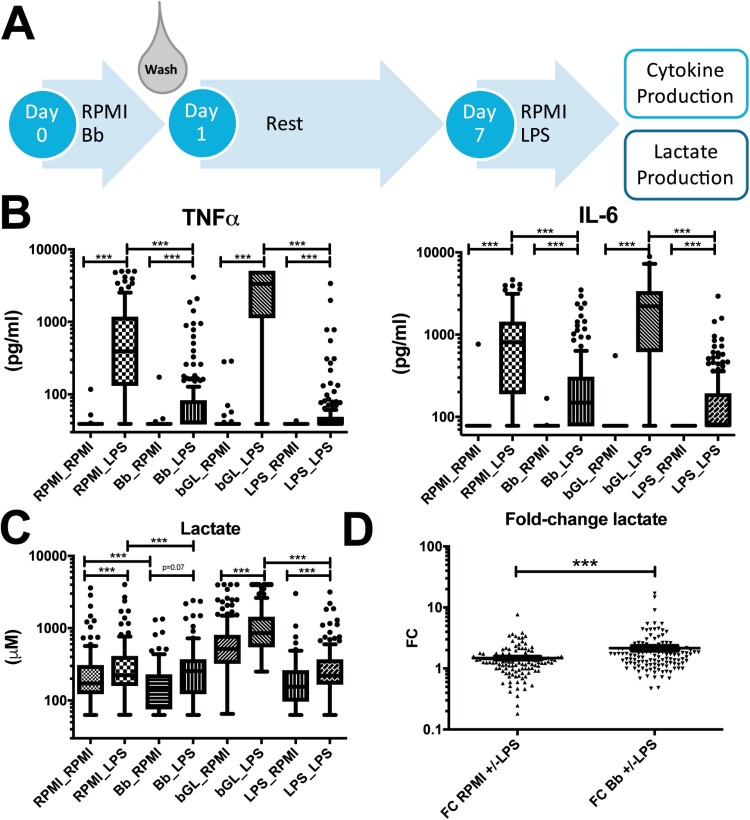
Inflammatory response of primary monocytes after exposure to *B. burgdorferi*. (**A)** Schematic representation of the trained immunity model. Primary monocytes are stimulated for 24 hour with medium control (RPMI), *B. burgdorferi* (Bb, MOI = 1), β-glucan (bGL, 2µL) or LPS (10 ng/ml). After 24 hour, the stimuli are washed away and cells are rested for six days, after which cells are restimulated with RPMI or LPS (10 ng/ml). After 24 hour, cell-free supernatants are collected for analysis of lactate- and cytokine production. (**B)** TNFα and IL-6 production in cell-free supernatants from monocytes of 151 healthy volunteers subjected to the trained immunity protocol described above. **(C)** Lactate concentration (µM) in cell-culture supernatant from primary monocytes of 120 healthy volunteers subjected to the trained immunity protocol described above. (**D)** Fold-change (FC) in lactate production after restimulation with LPS versus mock-treatment in primary monocytes previously exposed to *B. burgdorferi* or RPMI (*n* = 120). *** *P* < 0.0001 test with Wilcoxon matched-pairs signed rank test in panels B-D.

To determine whether exposure of primary human monocytes to *B. burgdorferi* influences their inflammatory response to a secondary, non-related stimulation, we used a well-characterized *in vitro* model of trained immunity/tolerance described previously [33]. Primary monocytes of 151 healthy volunteers were exposed for 24 hours to *B. burgdorferi*, fungal cell wall component β-glucan, or LPS; after 24 hours, cells were washed and rested for six days, after which cells were restimulated with the TLR4 ligand LPS (Fig. 1a). As expected, LPS restimulation induced significant production of TNFα and IL-6 in all conditions; however, cells that were previously exposed to *B. burgdorferi* produced significantly fewer cytokines than cells that had received mock-treatment (Fig. 1b). These findings were similar to those after prior LPS exposure, and in contrast to the increased cytokine production after exposure to β-glucan, as was previously described [34]. This indicates that *B. burgdorferi* exposure decreases the inflammatory potential of primary monocytes, similar to LPS-induced tolerance.

In line with the previous literature [19, 35], LPS restimulation induced increased lactate production in both RPMI as *B. burgdorferi* as first stimulation (Fig. 1c). Exposure to *B. burgdorferi* lowered baseline lactate production, although this could be rescued by restimulation with LPS. This suggests that *B. burgdorferi* stimulation induces a stronger upregulation of lactate production in response to LPS. Indeed, *B. burgdorferi*-treated cells had a statistically significantly higher fold-change (FC) in lactate production after LPS-restimulation than control cells (Fig.1d), however the actual difference between the groups was modest.

### Genetic variation in MFAP3L influences lactate production after *B. burgdorferi* exposure

**Figure 2:**
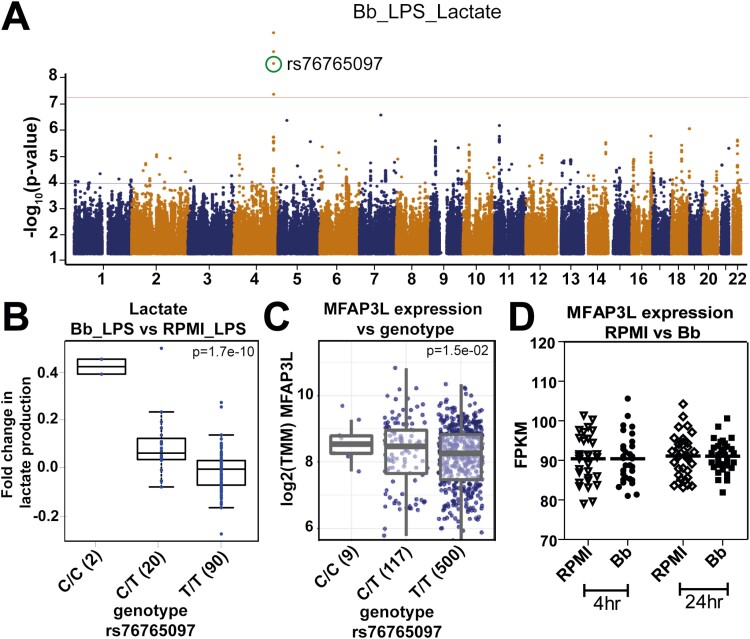
GWAS analysis indicates MFAP3L to be involved in lactate production in *B. burgdorferi*-exposed cells. (**A**) Manhattan plot of genome-wide QTL mapping of LPS-induced fold-changes in lactate production in primary monocytes previously exposed to *B. burgdorferi* (Bb). The upper horizontal line (red colour) represents the threshold for genome-wide significance (p<5x10-8). **(B)** LPS-induced fold change in lactate production in *B. burgdorferi*-exposed PBMCs obtained from individuals with different genotypes of SNP rs76765097. **(C)** Gene expression levels of MFAP3L, normalized by TMM method (Trimmed Mean of M values) in whole blood from healthy volunteers with different genotypes of rs76765097. P-values were calculated using a linear model with age and sex as covariates. (**D)** Relative gene expression of MFAP3L in PBMCs from healthy volunteers (*n* = 30) stimulated for 4 and 24 hour with *B. burgdorferi*. Data were extracted from a publicly available dataset through the NCBI Gene Expression Omnibus database, accession code GSE42606. Data are represented as median of fragments per kilobase of transcript per million mapped reads (FPKM).

For all parameters, significant inter-individual variation was observed in the responses to *B. burgdorferi* and LPS. To identify the source of this variation, a genome-wide association study (GWAS) was performed to identify quantitative trait loci (QTLs) affecting the fold-change in cytokine or lactate production induced by *B. burgdorferi*. One of the most significant QTLs was the single-nucleotide polymorphism (SNP) rs76765097 (*P* < 1.67 × 10^−10^), affecting LPS-induced lactate production (Fig. 2a). As previously mentioned, *B. burgdorferi*-exposure decreased baseline lactate production, while no difference was seen after LPS-restimulation between *B. burgdorferi*-exposed and unexposed monocytes (*P* = 0.07). However, individuals carrying the SNP exhibited a higher LPS-induced lactate production after *B. burgdorferi* exposure than without prestimulation (Fig. 2b). To elucidate what genes were affected by this SNP, expression QTL analysis was determined in the 500FG cohort [16], a separate cohort of healthy volunteers. Only one gene was found to be significantly affected, namely *microfibril-associated protein 3 like* (*MFAP3L*). As shown in Fig. 2c, individuals carrying the mutation had significantly higher levels of *MFAP3L* mRNA expression compared with wild-type individuals. Transcriptome analysis of *B. burgdorferi* exposed PBMCs of 36 healthy individuals [36] showed no upregulation of *MFAP3L* expression (Fig. 2d).

### MFAP3L influences lactate and cytokine production partially through ERK2

**Figure 3:**
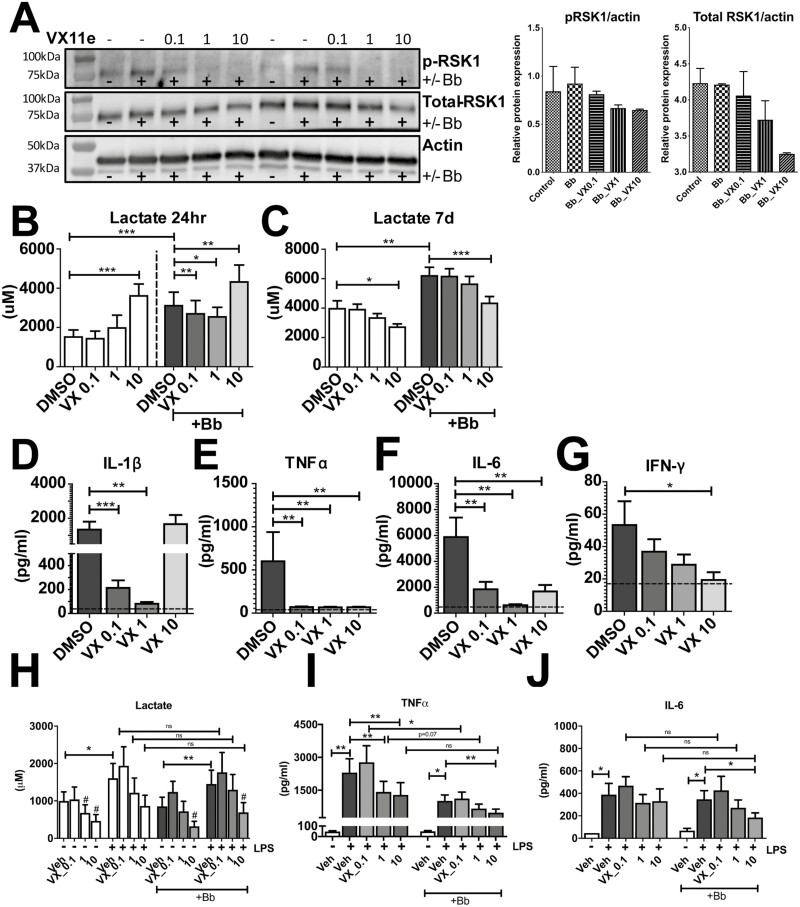
Effect of ERK2-inhibition on *B. burgdorferi*-induced lactate- and cytokine production. **(A)** Western blot of a downstream target of ERK pathway RSK1, both phosphorylated (p-RSK1) and the total amount RSK1 are represented in the first and second row, respectively. p-RSK1 is induced upon *B. burgdorferi* exposure and with ERK2 inhibitor VTX-11e is decreased in a dose-dependent manner. In the right panel the western blot data were quantified corrected for actin. Lactate production, measured in cell-free supernatants of PBMCs from healthy volunteers (*n* = 6) exposed for 24 hours **(B)** or 7 days **(C)** to *B. burgdorferi* or RPMI in the presence of different doses of the ERK2-inhibitor VTX-11e or vehicle control (DMSO). Cytokine production of IL-1β **(D)**, TNFα **(E)**, IL-6 **(F)**, and IFN-γ **(G)**, measured in cell-free supernatants of PBMCs from healthy volunteers (*n* = 6) exposed for 24hr (D-F) or 7 days (G) to *B. burgdorferi* or RPMI in the presence of different doses of the ERK2-inhibitor VTX-11e or vehicle control (DMSO). **(H)** Lactate production, measured in cell-free supernatants of primary monocytes from healthy volunteers (*n* = 6) exposed to *B. burgdorferi* for 24 hours in the presence of different doses of VTX-11e and, after a six-day resting period, restimulated for 24hr with LPS. # represents statistically significant lower lactate levels compared to vehicle control. Cytokine production of TNFα **(I)** or IL-6 **(J)**, measured in cell-free supernatants of primary monocytes from healthy volunteers (*n* = 6) exposed to *B. burgdorferi* for 24 hours in the presence of different doses of VTX-11e and, after a six-day resting period, restimulated for 24hr with LPS. *P < 0.05, **P < 0.01, ***P < 0.0001 tested with Wilcoxon matched-pairs signed rank test for panels B-J.

eQTL analysis determined that a genetic variation leading to increased *MFAP3L* expression was critical for lactate production in *B. burgdorferi*-stimulated primary monocytes. To validate these findings, we attempted to elucidate the functional role of the MFAP3L protein in *B. burgdorferi* stimulations. As MFAP3L protein was reported to be partly located at the level of the cell membrane, we attempted to block MFAP3L activation using a neutralizing antibody. However, the addition of a polyclonal anti-MFAP3L antibody did not affect *B. burgdorferi*-induced cytokine nor lactate production in either a direct PBMC stimulation or the trained immunity model (Supplimentary Fig. S1). This suggested that MFAP3L acts mainly intracellularly. An earlier study suggested that the protein exhibits kinase activity and activates the serine/threonine kinase ERK2 [37]. We investigated the role of the signaling molecule ERK2, using the pharmacological ERK2 inhibitor VTX-11e in a PBMC model of *B. burgdorferi* infection. As VTX-11e inhibits ERK2 in an ATP-competitive manner, without affecting phosphorylation levels, we confirmed the effect of the inhibitor by analyzing the phosphorylation levels of one of its main downstream targets, RSK1. As shown in Fig. 3a, levels of p-RSK1 were induced after *B. burgdorferi* stimulation and dose-dependently decreased by VTX-11e. As expected, exposure to *B. burgdorferi* significantly increased lactate production in PBMCs, both after 24 hours and 7 days (Fig. 3b-c). Inhibition of ERK2 affected lactate production in a dose- and time-dependent manner. After 24 hours, low- (*P* = 0.0029) and moderate ERK2-inhibition (*P* = 0.0322) decreased *B. burgdorferi*-induced lactate production, while high-dose ERK2-inhibition increased lactate production. This induction by high-dose ERK2-inhibition may be the result of nonspecific effects of the inhibitor. After 7 days of *B. burgdorferi* stimulation, the highest concentration of ERK2-inhibition showed a decrease in lactate production. Taken together, these data indicate that ERK2 plays an essential role in regulating lactate production in response to *B. burgdorferi*.

As lactate production is a measure of the rate of glycolysis, and glycolysis has previously been shown to be important for cytokine production [16, 38], we set out to determine whether ERK2 also plays a role in *B. burgdorferi*-induced cytokine production. Noteworthy, ERK2-inhibition exhibited a similar effect on IL-1β production as was seen for lactate production, with the low- and intermediate dose decreasing cytokine production and the high dose showing a trend toward increased IL-1β release (Fig. 3d). In contrast, for TNFα and IL-6, all doses significantly downregulated cytokine production. After 7 days of incubation, a dose-dependent decrease in the production of IFN-γ could still be observed, even though only the highest dose reached statistical significance.

To rule out that any effects of the inhibitor were due to cytotoxicity, the concentrations of lactate dehydrogenase (LDH) were measured in cell-free supernatants of all conditions tested. As seen in Suppl. Fig. 2, LDH release was comparable in VTX-11e treated cells and *B. burgdorferi*-exposed cells. Taken together, ERK2-inhibition was found to decrease cytokine production after 24 hours and 7 days of *B. burgdorferi* stimulation. These data are also supporting the role of glycolysis in the inflammatory response, as lactate production correlated well with cytokine production.

As the MFAP3L-ERK2 pathway was originally identified in a trained-immunity model, we also investigated whether ERK2 played a role in the long-term effects of *B. burgdorferi* stimulation on monocytes. Primary monocytes were pretreated with the ERK2 inhibitor for 1 hour, exposed to *B. burgdorferi* for 24 hours and then cultured and restimulated according to the trained immunity protocol as described above.

As shown in Fig. 3h, LPS restimulation increased lactate production in both untreated and *B. burgdorferi-*stimulated cells. Again, addition of the ERK2-inhibitor resulted in decreased lactate (Fig. 3h), as well as TNFα (Fig. 3i) and IL-6 (Fig. 3j) production, in a dose-dependent manner. However, when comparing lactate and cytokine production between *B. burgdorferi* exposed and unstimulated monocytes before restimulation with LPS, only TNFα production decreased significantly. Together, these data suggest that ERK2 indeed plays a role in *B. burgdorferi*-induced lactate and cytokine production and may be one mechanism by which MFAP3L regulates lactate production.

### The role of (MFAP3L)-activated platelets in the host response against *B. burgdorferi*

**Figure 4:**
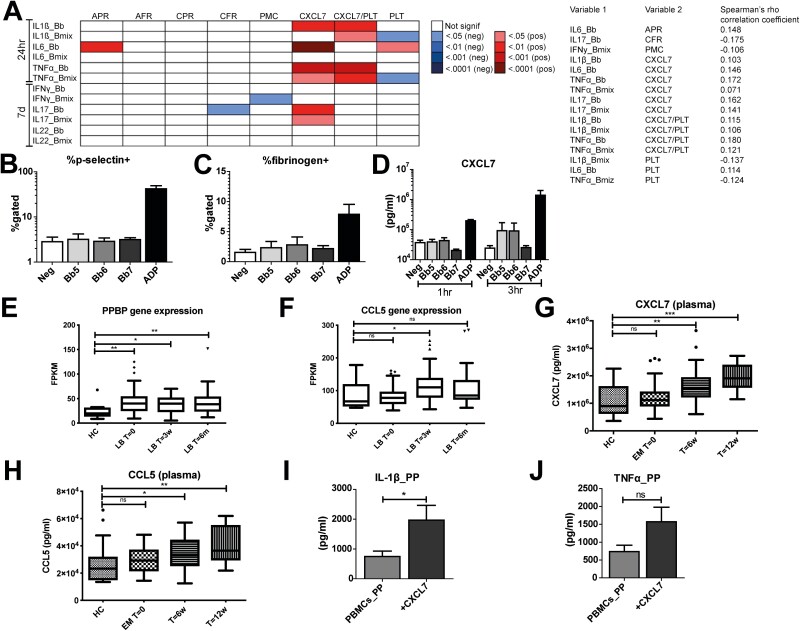
Role of platelets in *B. burgdorferi*-induced cytokine production. (**A**) FDR-corrected *P*-values of correlations between circulating platelet parameters and cytokine production by PBMCs stimulated ex-vivo in a cohort of 489 healthy participants. Platelet markers include ADP- and CRP-XL-induced platelet P-selectin (APR and CPR); platelet-fibrinogen binding to either ADP or CRP-XL (AFR and CFR); platelet-monocyte complexes (PMC); CXCL7 levels, CXCL7 levels corrected for platelet count (CXCL7/PLT); total platelet count (PLT). The correlation coefficients of the statistically significant correlations are provided, the non-significant correlation coefficients are not displayed. (**B-C**) Percentage of P-selectin- and fibrinogen positive platelets (*n* = 6 healthy volunteers), determined by flow cytometry after 3hr stimulation with increasing doses of *B. burgdorferi* or the positive control ADP. Bb5/6/7 = stimulation with *B. burgdorferi* 10^5/6/7 spirochetes/ml. (**D)** CXCL7 secretion in cell-free supernatants of platelets from healthy volunteers (*n* = 6) stimulated for 3hr with increasing doses of *B. burgdorferi* or the positive control ADP. Expression of the gene PPBP (CXCL7) **(E)** and CCL5 **(F)** in physician-confirmed LB patients (including both EM and multiple EM) at different time points (*n* = 29) compared to healthy controls (*n* = 13). Data were extracted from the NCBI GEO database, accession number GSE63085. Data are represented as median±IQR of fragments per kilobase of transcript per million mapped reads (FPKM). The concentration of both CXCL7 **(G)** and CCL5 **(H)** were elevated in the plasma of 82 EM patients, including 14 patients with multiple timepoints, compared to 19 healthy controls. Cytokine production of IL-1β **(I)** and TNFα **(J)** in platelet-poor (PP) PBMCs from healthy volunteers (*n* = 6) stimulated for 24hr with *B. burgdorferi* in the presence of rhCXCL7 (20µg/ml). Ns = not statistically significant, *P < 0.05, **P < 0.01, ***P < 0.0001 calculated using independent-samples Mann-Whitney *U* test for comparing HC and LB patient cohort and Wilcoxon matched-pairs signed ranked test for paired testing when comparing LB patient different time points in panels E-H. Wilcoxon matched-pairs signed ranked test was used in panels I and J.

Having confirmed the link between ERK2 and *B. burgdorferi*-induced production of cytokine and lactate, we moved on to investigate the second suggested function of *MFAP3L*. The gene network prediction model [30, 39, 40] suggested that *MFAP3L* plays a role in platelet aggregation and platelet activation. Indeed, platelet function has been linked to MFAP3L expression [41]. Platelets are increasingly being appreciated for their role in inflammation [42, 43]. To investigate whether platelets play a role in the context of *B. burgdorferi* infection, we investigated the 500FG cohort in which several functional parameters of the immune system have been mapped [21, 44]. For this analysis, we assessed several markers of platelet activation, such as surface expression of P-selectin, fibrinogen-binding to the activated α_IIb_β_3_ integrin, formation of platelet-monocyte complexes, and circulating levels of the α-granule protein CXCL7. These markers were correlated to *ex vivo* cytokine production by PBMCs to identify platelet factors that influenced cytokine production induced by *B. burgdorferi.* Noteworthy, especially CXCL7 was found to show a statistically significant correlation to *B. burgdorferi*-induced production of IL-1β and TNFα, even after correction for total platelet count (Fig. 4a). IL-6 and IL-17 also showed a highly significant correlation to CXCL7, but this appeared to be due to the platelet count, as these correlations were absent after correction for the platelet count. Interestingly, *B. burgdorferi*-induced IL-1β and TNFα showed a negative correlation with total platelet count. This suggests that platelets and platelet-derived proteins might play a dual role in regulating cytokine production in response to *B. burgdorferi*.

To validate the role of platelets in *B. burgdorferi* infection, we first determined whether the spirochete was able to independently activate platelets. Platelet-rich plasma (PRP) from healthy volunteers was exposed for 1 and 3 hours to *B. burgdorferi* and adenosine diphosphate (ADP) as a positive control. After incubation, flow cytometry was performed to determine the membrane expression of the α granule platelet marker P-selectin and binding of fibrinogen to the activated integrin α_IIb_β_3_ on the platelet membrane. As shown in Fig. 4b, while ADP-stimulation substantially increased the percentage of P-selectin and fibrinogen-positive platelets, no such effect could be seen for *B. burgdorferi*. To determine whether *B. burgdorferi* may specifically lead to the secretion of CXCL7, we analyzed the CXCL7 production in the cell-free supernatants of the stimulated samples. In line with the findings shown in Fig. 4b and c, no change in CXCL7 levels was observed following *B. burgdorferi* exposure, while a substantial increase in CXCL7 levels was seen after incubation with the positive control ADP. Taken together, these data indicate that short exposure of *B. burgdorferi* is not able to activate platelets directly *in vitro*. It could be that an interaction of monocytes, platelets, and *B. burgdorferi* is needed to activate platelets *in vivo.*

As we found the strongest correlation between CXCL7 levels and cytokine responses, we further investigated this process. To consider the involvement of other cell types in the interaction with platelets, we investigated the expression of CXCL7 *in vivo*. For this, we made use of transcriptome data of PBMCs from patients with erythema migrans, obtained from a previously published dataset [45]. Interestingly, the expression of pro-platelet basic protein (*PPBP*), the gene encoding for CXCL7, was significantly increased in unstimulated PBMCs of LB patients compared to healthy controls (Fig. 4e). Strikingly, expression remained elevated up to six months after diagnosis. Considering the high expression levels, it is likely that the mRNA expression is derived from platelets, which are known to be abundantly present in PBMC populations. Indeed, we found an elevated plasma concentration of CXCL7 in erythema migrans (EM) patients at both 6 and 12 weeks after EM diagnosis and following antibiotic treatment compared with healthy controls (Fig. 4g). We could confirm that this findings in serum of different cohort of EM patients (Supplimentary Fig. S5). Interestingly, the gene expression (Fig. 4f) and plasma concentration (Fig. 4h) of a different platelet-derived chemokine, chemokine (C-C motif) ligand 5 (CCL5), was also elevated in EM patients, providing additional support for a role of platelets in the pathogenesis of EM. These platelet-derived markers are sensitive for preanalytical factors, however. While we found comparable platelet counts in whole blood of the healthy controls and EM patients, we observed an increased immature platelet fraction (IPF) in whole blood of EM patients compared with healthy controls (Supplimentary Fig. S6b). Immature platelets have been shown to be more biochemically active than their mature counterparts [46]. These findings support the notion of the involvement of platelets in the human host immune response against *B. burgdorferi.*

Having confirmed that CXCL7 is upregulated in the context of *B. burgdorferi* infection *in vivo*, we determined whether it would indeed play a role in the inflammatory response. First, we attempted to inhibit the action CXCL7 with a neutralizing antibody. Even though ELISA analysis indicated that the antibody indeed bound its target, as lower levels of CXCL7 were detected (Supplimentary Fig. S3a), no effects could be detected on cytokine or lactate production (Supplimentary Fig. S3b,c). This indicates either that CXCL7 has no effect on cytokine production or that binding of the antibody to its target does not affect its signaling function.

To elucidate this, we determined the effect of adding recombinant human CXCL7 (rhCXCL7). As steady-state levels of CXCL7 in a PBMC model are already high, due to a large number of platelets present, we first lowered the number of platelets in our PBMC population using extra washing steps. After this, we pre-incubated cells for 1 hour with rhCXCL7 after which cells were stimulated with *B. burgdorferi*. As shown in Fig. 4i and j, the addition of rhCXCL7 significantly increased the production of both IL-1β and TNFα, suggesting that it is indeed involved in the inflammatory response. A role for LPS-contamination was excluded by adding a 10-fold excess of the TLR4 antagonist *Bartonella quintana* LPS (Supplimentary Fig. S4).

Taken together, these data show that even though platelets are not directly activated by *B. burgdorferi*, platelet-derived molecules such as CXCL7 play an active role in inducing an inflammatory response.

## Discussion

This study shows that exposing human monocytes to *B. burgdorferi* decreased their capacity to respond to a secondary stimulus. In addition, we found a genetic variant affecting the expression of *MFAP3L* that influences *B. burgdorferi*-induced lactate production. Furthermore, we provide evidence that the downstream effects of *MFAP3L* might be partially mediated by ERK2 and through platelet activation. Finally, we provide new data suggesting that platelets may play an active role in the inflammatory response against *B. burgdorferi*, possibly mediated by the platelet-derived chemokine CXCL7.

The concept of trained immunity has been proposed during the last years, initiated from the finding that monocytes respond stronger when they have been previously exposed to certain stimuli [47]. Mechanistically, this has been explained by intracellular metabolic changes including an increased glycolytic rate [19]. As our previous findings also showed an increased glycolytic rate after *B. burgdorferi*-stimulation [16], we hypothesized that the spirochete might induce trained immunity as well. In contrast, we found that initial exposure to *B. burgdorferi* decreased cytokine production by monocytes, like the well-known LPS-induced innate immune tolerance [48, 49]. Correspondingly, we found that *B. burgdorferi* exposure decreased long-term baseline lactate production in comparison to control cells. This was somewhat surprising, as we know from previous studies that acute stimulation with the pathogen increases lactate production [16]. This suggests the presence of a negative feedback mechanism that downregulates late lactate production after the initial increase. In support of this, *B. burgdorferi*-exposed cells were able to restore their lactate production to control levels when stimulated with LPS.

To further unravel the mechanism behind these responses, GWAS analysis was performed to find genetic variants affecting LPS-induced lactate or cytokine production in *B. burgdorferi-*exposed cells. The most significant correlation was found for an SNP affecting lactate production; individuals carrying this mutation produced more lactate when previously exposed to *B. burgdorferi*. This suggests that the mutation either prolongs the *B. burgdorferi*-induced acute state of increased glycolysis or affects the cell’s metabolic reactivity. To further elucidate this, eQTL analysis of whole blood was performed in a separate cohort to identify genes that were affected by the variant. The only gene found to be significantly affected by the variant was *MFAP3L*. *MFAP3L* is a protein-coding gene and it has been suggested to participate in the nuclear signaling by ERK2/MAPK1 and EGFR [37]. Supporting the link between MFAP3L, ERK2, and glycolysis, the addition of a specific ERK2 inhibitor decreased lactate production in both a PBMC and a monocyte training model. This is in accordance with previous studies showing that ERK2 is important for the induction of glycolysis [50, 51]. Furthermore, ERK2 inhibition substantially decreased cytokine responses induced by *B. burgdorferi*, both in the acute stimulation as in the trained immunity model. These findings are also supported by previous literature, as ERK2 has been shown to play a role in cytokine production by multiple cell types in response to *B. burgdorferi* [52, 53].

In addition to ERK2, gene ontology analysis suggested that *MFAP3L* might have a function in the activation and degranulation of platelets. The role of platelets in innate immunity is increasingly being recognized. Platelets have been shown to express functional TLRs [54–56] and can recognize and respond to pathogens. In addition, platelets have been shown to directly interact with neutrophils [57, 58], dendritic cells [59, 60], and monocytes [61, 62] thereby regulating the inflammatory response. Especially the latter is noteworthy in this context; platelets were shown to differentially affect monocyte responses based on the encountered antigen [43, 63].

So far, only few studies have studied the role of platelets in *B. burgdorferi* infection, and there are some reports concerning platelets in the pathogenesis of relapsing fever by *Borrelia recurrentis* [64, 65]. It has been hypothesized that *B. burgdorferi*-induced vascular damage [66, 67] is followed by platelet activation and aggregation, leading to binding of *B. burgdorferi* to platelets, mediated by integrin α_IIb_β_3_, yet only if these platelets have been previously activated [68–70]. Virulent *B. burgdorferi* could bind platelets more efficiently than non-virulent spirochetes [69, 70], suggesting that platelet binding could be related to infectivity. This binding could be mediated through *B. burgdorferi* surface protein p66 [71].

We found that *B. burgdorferi* was unable to activate platelets in the absence of other cell types. Nevertheless, our data indicated a strong positive correlation between the circulating platelet-derived protein CXCL7 and *ex vivo* cytokine production in healthy volunteers. In contrast, a negative correlation was seen between TNFα and IL-1β production and total platelet count. This is in line with a previous study that found that platelets negatively affected cytokine production induced by Pam3Cys, another TLR2 ligand [43]. IL-6 appeared to be an exception in our analysis, showing a positive correlation with platelet count, although this might be due to the relationship with CXCL7 [72, 73]. Subsequently, we found that expression of *PPBP*, the gene encoding for CXCL7, was significantly increased in PBMCs from EM patients, even up to six months after baseline. Next, we found that CXCL7 and CCL5 plasma concentrations were elevated in EM patients compared to healthy controls. In EM patients, CXCL7 and CCL5 were raised after six and even twelve weeks following the start of antibiotic treatment, but, interestingly, not at the time of diagnosis. The unaffected CXCL7 plasma concentration at baseline could be an effect of immune evasion strategies of *B. burgdorferi* s.l [74]. Persistently elevated circulating CXCL7 and CCL5 concentrations can be explained in multiple ways. First, training of megakaryocytes [75, 76]. Second, persistent or recurring degranulation of platelets in response to *B. burgdorferi.* Third, of the 94 EM patients depicted in Fig. 4, 88 (93.6%) received doxycycline, which interferes with anti-inflammatory macrophage differentiation and can inhibit matrix metalloproteinases (MMPs) [77, 78]. In addition, broad-spectrum antibiotics affect the host microbiome and can alter host immunity leading to enhanced inflammatory signatures [79].

Upregulation of *PPBP* in PBMCs was also described in a recent study in patients with multiple EM, both at baseline and after 1 month, but not after 6 months [80]. In addition, CXCL7 gene expression was upregulated in dendritic cells in response to *B. garinii* stimulation compared with unstimulated and LPS-stimulated cells [81], which might be due to platelet contamination however. During a different spirochetal infection, neurosyphilis, elevated CXCL7 concentrations in the cerebrospinal fluid were reported [82]. Finally, we found that the addition of recombinant CXCL7 increased cytokine production in an *in vitro* PBMC model of *B. burgdorferi* stimulation, supporting an active role for CXCL7 in the inflammatory response to *B. burgdorferi.*

A limitation of this study was that plasma from EM patients was only available after overnight shipment. Twenty-four hours of storage of platelets causes platelet aggregates and the release of platelet-derived platelet factors (PF), such as PF 4 [83]. We corrected for this by matching the time until centrifugation of EM patients with healthy controls. It would be interesting to measure CXCL7 and CCL5 in freshly obtained patient plasma to account for spontaneous degranulation of platelets. To address this problem, we measured CXCL7 in serum, which was directly obtained after blood collection and we could confirm increased circulating CXCL7 in EM patients. Moreover, the correlation of CXCL7 with *B. burgdorferi*-induced cytokine production displayed in Fig. 4a was found in freshly isolated platelets and freshly isolated PBMCs [84]. Considering all the above, CXCL7 might not be a causal factor in *B. burgdorferi-*induced cytokine production, but platelet degranulation in general. For instance, the gene of another platelet α-granule, PF4, was shown to be upregulated in 2 transcriptome studies of EM patients [45, 80].

Another well-known platelet factor, CCL5, was found to be elevated in the plasma of EM patients as well. CCL5, also known as Regulated upon Activation Normal T-Cell Expressed and Secreted (RANTES), is produced by several cell types and attracts not only T cells, but also other immune cells such as dendritic cells, eosinophils, and NK cells [85, 86]. Interestingly, tick saliva inhibits the binding of CCL5, to its receptors by chemokine binding proteins, called Evasins, that have anti-inflammatory properties in animal models [87]. For instance, in Lyme neuroborreliosis [88] and Lyme arthritis [89] CCL5 mRNA expression and production were increased following *B. burgdorferi* exposure.

CXCL7 belongs to the CXC chemokine family and has indeed been shown to function as a chemoattractant for neutrophils [90] but also monocytes [91]. In addition, CXCL7 has been reported to play a role in angiogenesis, cell proliferation, and antimicrobial defense [92–96]. Noteworthy, CXCL7 has also been reported to be involved in regulating the expression of glucose transporters and thereby glucose uptake in connective tissue cells [97]. CCL5 is involved in glucose metabolism as well [98], as it regulates glucose uptake in activated T cells to facilitate chemotaxis [99]. It is tempting to speculate that increased CXCL7 and CCL5 levels may be a mechanism by which increased *MFAP3L* expression affects lactate production. ERK2 may also have a mediating role in this process, as ERK2 has proven to be active in platelets [100] and play a role in platelet activation and degranulation [101, 102]. Taken together, we propose a model in which increased expression of *MFAP3L* enables activation of ERK2, which promotes platelet degranulation and release of CXCL7 and CCL5 in response to *B. burgdorferi* which may affect glucose uptake and lactate production in monocytes. Our *in vitro* data indicate that this pathway also substantially affects cytokine production, which is not surprising, considering the strong link between glucose metabolism and inflammation [16].

In summary, this study indicates that exposure of monocytes to *B. burgdorferi* decreases their inflammatory potential, suggestive of innate immune tolerance. This may on the one hand be an immune evasion strategy of the spirochete, and speculatively, it may also increase the susceptibility of the host to other infections. Further studies are needed to determine the clinical consequences of this effect. Furthermore, we identified the ERK2 pathway and platelet activation not only as mediators in *B. burgdorferi*-induced phenotypical changes in monocytes, but also as general players in the host response against this pathogen. Considering the important role of the host inflammatory response in LB pathogenesis, these findings may prove useful for explaining the variability in clinical phenotype seen in LB.

## Supplementary Material

uxac073_suppl_Supplementary_Figures

## Data Availability

The authors confirm that the data supporting the findings of the present study are available from the corresponding author (Leo Joosten) upon reasonable request.

## Abbreviations

200FG: 200 Functional Genomics cohort
500FG: 500 healthy individuals of Western-European ancestry from the Human Functional Genomics Project
ARLA: antibiotic-refractory Lyme arthritis
ATP: adenosine triphosphate
*B. burgdorferi*: *Borrelia burgdorferi*
BSK: Barbour-Stoenner-Kelley
CCL5: C-C motif ligand 5
CXCL7: C-X-C motif ligand 7
EM: erythema migrans
ERK2: extracellular signal-regulated kinase 2
GWAS: genome-wide association study
IFN-γ: interferon-gamma
IL: interleukin
IPF: immature platelet fraction
LB: Lyme borreliosis
LDH: lactic acid dehydrogenase
LPS: lipopolysaccharide
MFAP3L: microfibril-associated protein 3 like
PBMC: peripheral blood mononuclear cell
PPBP: pro-platelet basic protein
PTLDS: Post-Treatment Lyme Disease Syndrome
RANTES: regulated upon activation normal T cell expressed and secreted
RPMI: Roswell Park Memorial Institute
RSK1: ribosomal S6 kinase 1
s.l.: sensu lato
s.s.: sensu stricto
STARI: southern tick-associated rash illness
TLR4: toll-like receptor 4
TNF: tumor necrosis factor.
