# Supplementary material for: Identifying platelet-derived factors as amplifiers of B. burgdorferi-induced cytokine production
Source: Clin Exp Immunol. 2022 Aug 24;210(1):53–67. doi: 10.1093/cei/uxac073 (PMC9585555; doi:10.1093/cei/uxac073)
Supplement: uxac073_suppl_Supplementary_Figures [file uxac073_suppl_supplementary_figures.docx]

**Supplementary information**

1. Figure S1. Effect of MFAP3L neutralization on *B. burgdorferi* induced lactate- and cytokine production
2. Figure S2. LDH-release after treatment with increasing doses of VTX-11e
3. Figure S3. Effect of CXCL7 neutralization on *B. burgdorferi*-induced lactate- and cytokine production
4. Figure S4. TLR-4 blocking does not affect the induction of cytokines by CXCL7
5. Figure S5. CXCL7 levels in serum of EM patients elevated compared to healthy controls
6. Figure S6. Platelet counts and IPF in EM patients compared to healthy individuals

**Supplementary information**

**Figure S1. Effect of MFAP3L neutralization on *B. burgdorferi* induced lactate- and cytokine production
A.** Lactate production, measured in cell-free supernatants of PBMCs from healthy volunteers (n=6) exposed for 24hr or 7 days to *B. burgdorferi* or RPMI in the presence of anti-human MFAP3L or isotype control antibody. **B.** Cytokine production of IL-1β, TNFα, and IL-6, measured in cell-free supernatants of PBMCs from healthy volunteers (n=6) exposed for 24hr to *B. burgdorferi* or RPMI in the presence of anti-human MFAP3L or isotype control antibody. **C.** Lactate production, measured in cell-free supernatants of primary monocytes from healthy volunteers (n=6) exposed to *B. burgdorferi* for 24hr in the presence of different doses of anti-human MFAP3L or isotype control antibody and, after a six-day resting period, restimulated for 24hr with LPS. **D.** Cytokine production of TNFα or IL-6, measured in cell-free supernatants of primary monocytes from healthy volunteers (n=6) exposed to *B. burgdorferi* for 24hr in the presence of different doses of anti-human MFAP3L or isotype control antibody and, after a six-day resting period, restimulated for 24hr with LPS.

**Figure S2. LDH-release after treatment with increasing doses of VTX-11e**Levels of lactate dehydrogenase (LDH) were measured in cell-free supernatants of PBMCs from healthy volunteers (n=5) exposed for 24hr or 7 days to *B. burgdorferi* or RPMI in the presence of increasing doses of VTX-11e. Positive control represents well with all cells lysed to induce maximal LDH release. Data are represented as median and interquartile range (IQR) of optical density (OD).

**Figure S3. Effect of CXCL7 neutralization on *B. burgdorferi*-induced lactate- and cytokine production
A.** CXCL7 levels, measured in cell-free supernatants of PBMCs from healthy volunteers (n=6) exposed for 24hr to *B. burgdorferi* or RPMI in the presence of anti-human CXCL7 or isotype control antibody. **B.** Cytokine production of IL-1β and TNFα, measured in cell-free supernatants of PBMCs from healthy volunteers (n=6) exposed for 24hr to *B. burgdorferi* or RPMI in the presence of anti-human CXCL7 or isotype control antibody. **C.** Lactate production, measured in cell-free supernatants of PBMCs from healthy volunteers (n=6) exposed to *B. burgdorferi* for 24hr in the presence of different doses of anti-human CXCL7or isotype control antibody.

**Figure S4. TLR-4 blocking does not affect the induction of cytokines by CXCL7**
Cytokine production of IL-1β and TNFα, measured in cell-free supernatants of PBMCs from healthy volunteers (n=3) exposed for 24hr to *B. burgdorferi* or RPMI in the presence of recombinant human CXCL7 with or without the addition of the TLR-4 antagonistic LPS from *Bartonella quintana* (Bart.LPS)

**Figure S5. CXCL7 levels in serum of EM patients elevated compared to healthy controls**

Circulating CXCL7 serum levels in EM patients were elevated compared to healthy controls at most time points up to 12 weeks following diagnosis. This confirms the findings in heparin plasma. Ns = not statistically significant, * p < 0.05 calculated using independent-samples Mann-Whitney U test for comparing HC and LB patient cohort

**Figure S6. Platelet counts and IPF in EM patients compared to healthy individuals**

Complete blood cell counts of whole blood were analyzed using the Sysmex hematological analyzer. **A.** Platelet count in whole blood was similar between healthy controls and EM patients. **B.** Immature platelet fraction (IPF) was elevated in EM patients compared to healthy controls. The data are represented with median and interquartile range. ns = not statistically significant, ** p < 0.01, *** p < 0.0001 calculated using independent-samples Mann-Whitney U test for comparing HC and LB patient cohort and Wilcoxon signed-ranked test for paired testing when comparing LB patient time points in both panels.


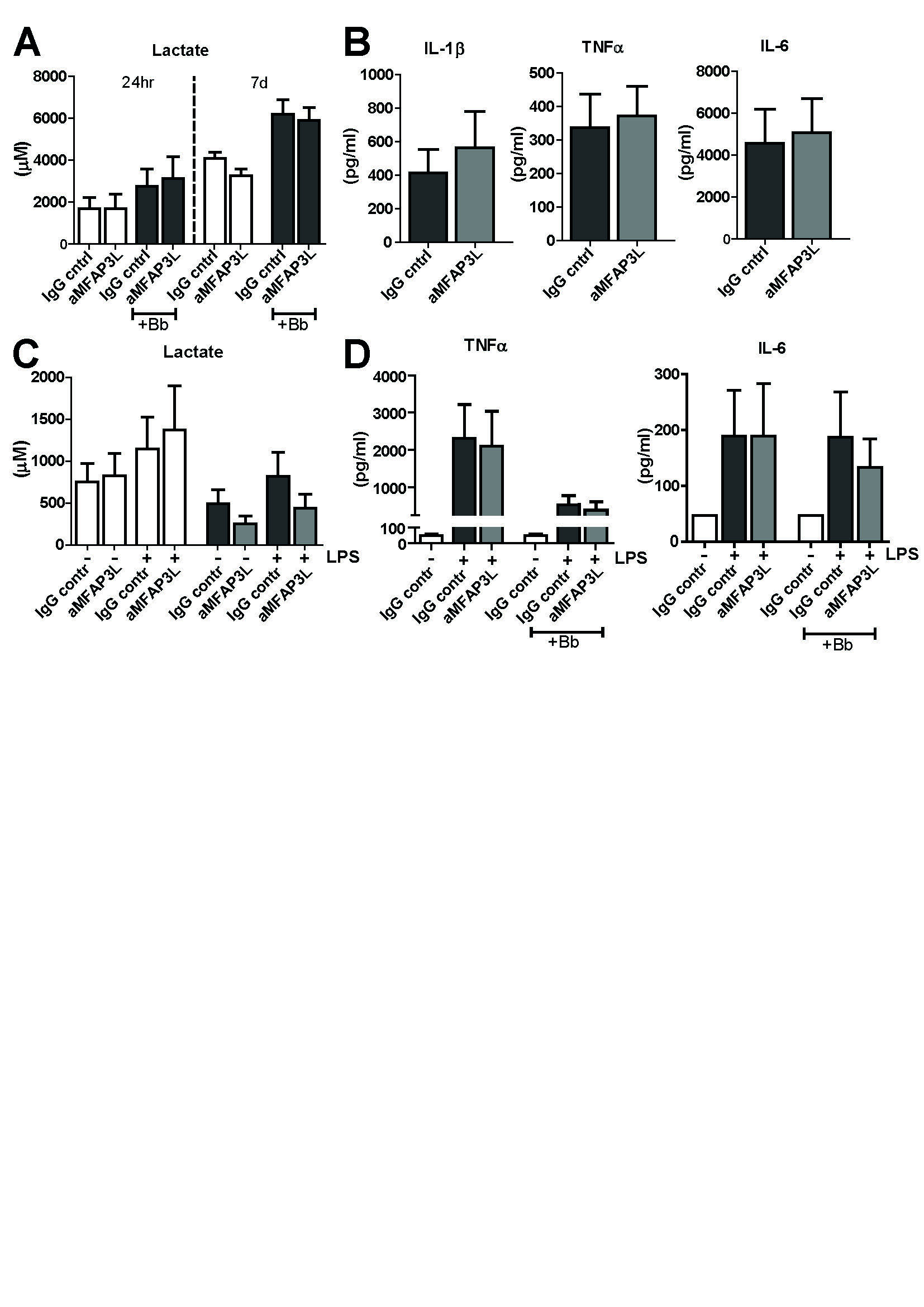


**Figure S 1,** related to Fig. 3

**
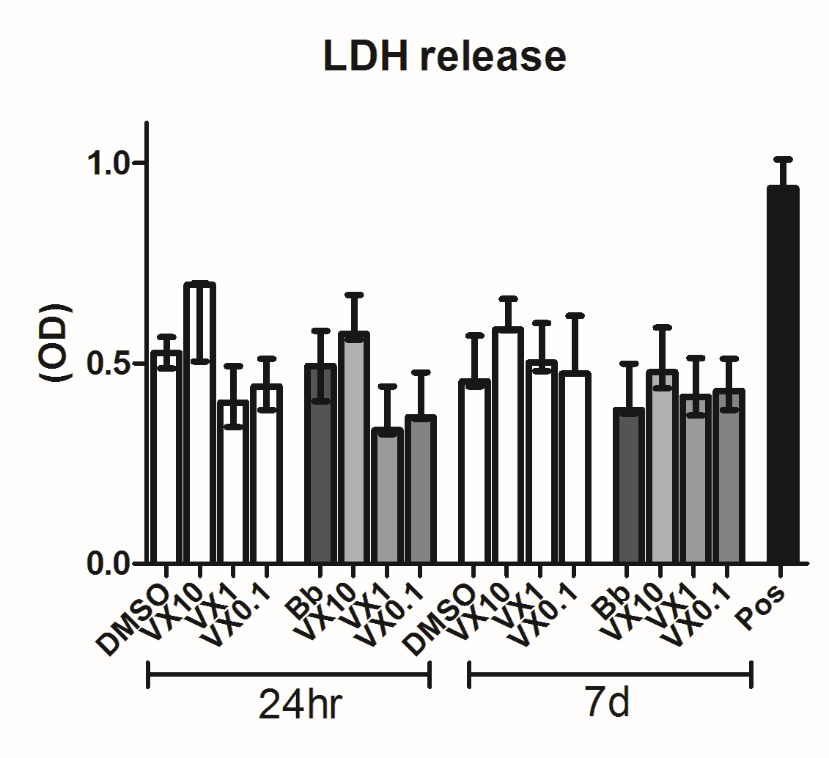
**

**Figure S2,** related to Fig. 3


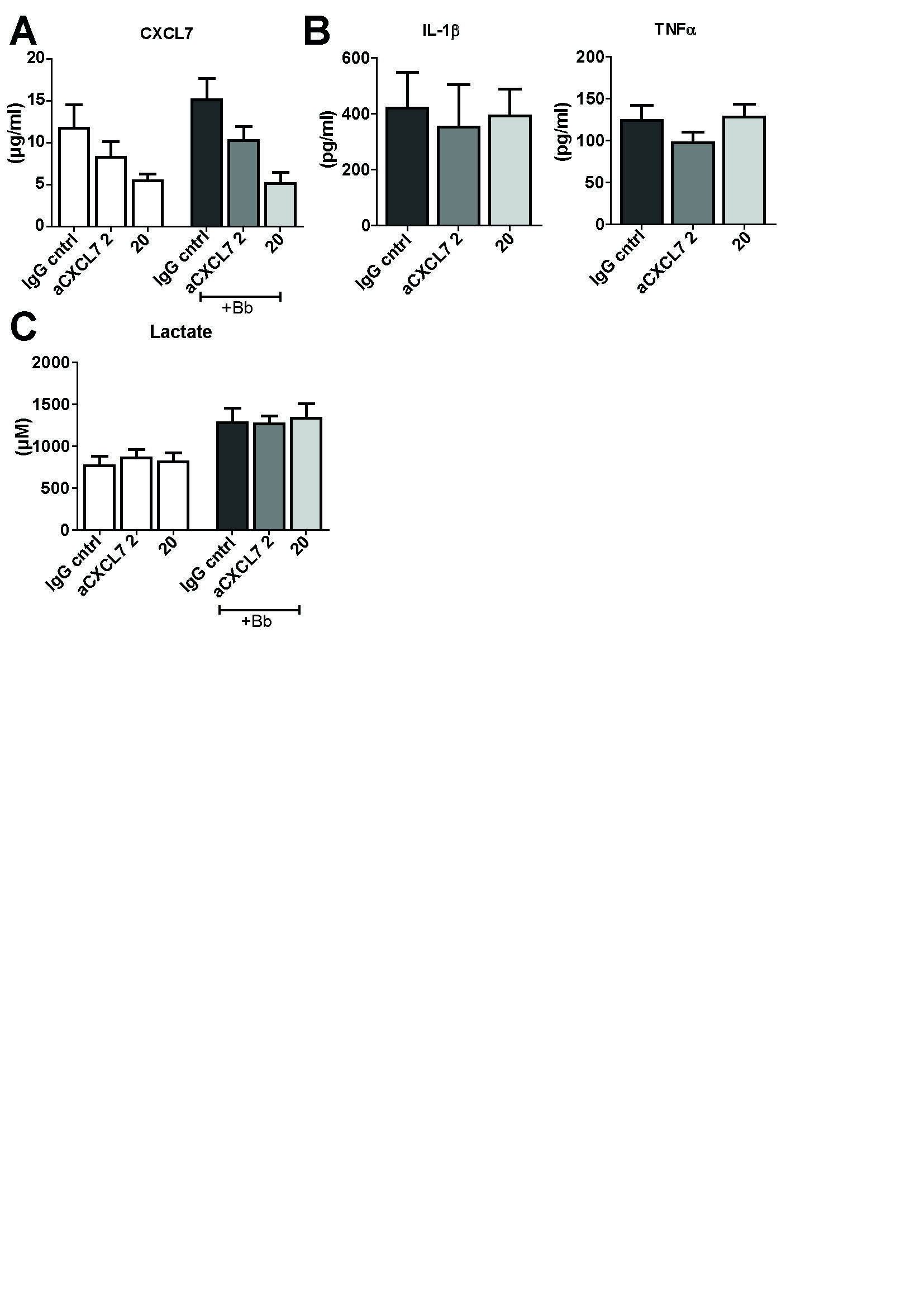


**Figure S3,** related to Fig. 4


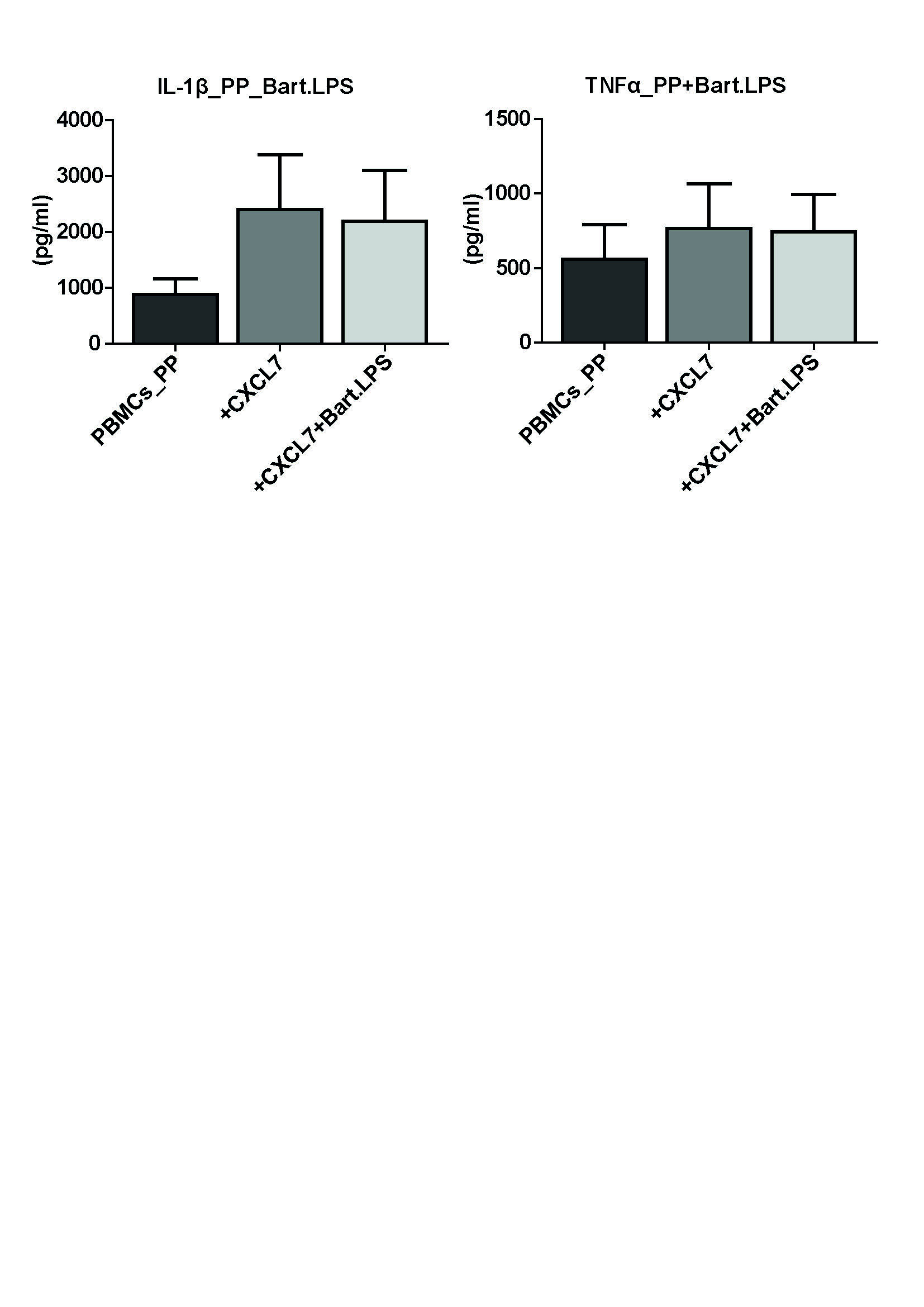


**Figure S4,** related to Fig. 4

*
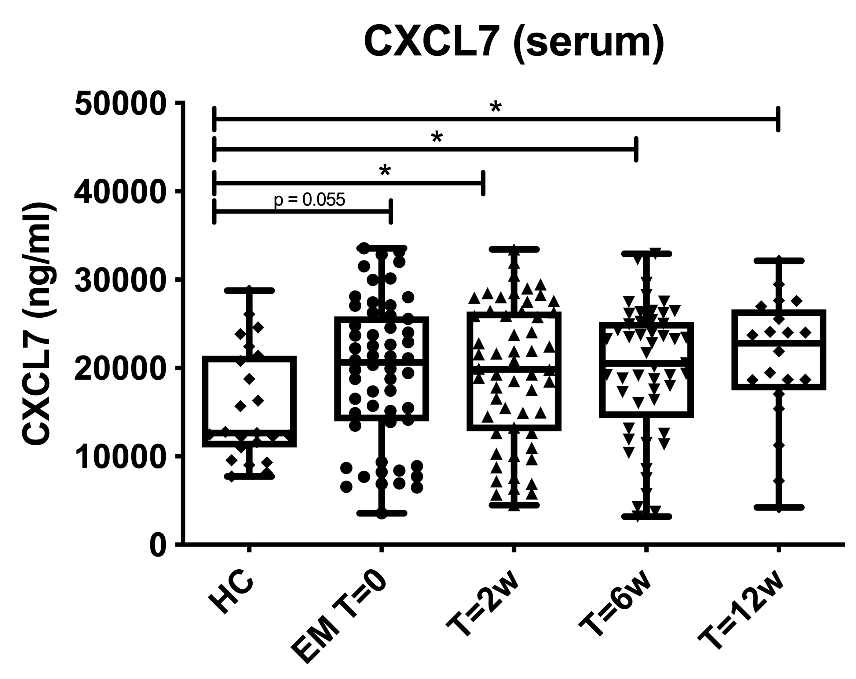
*

**Figure S5**, related to Fig. 4.


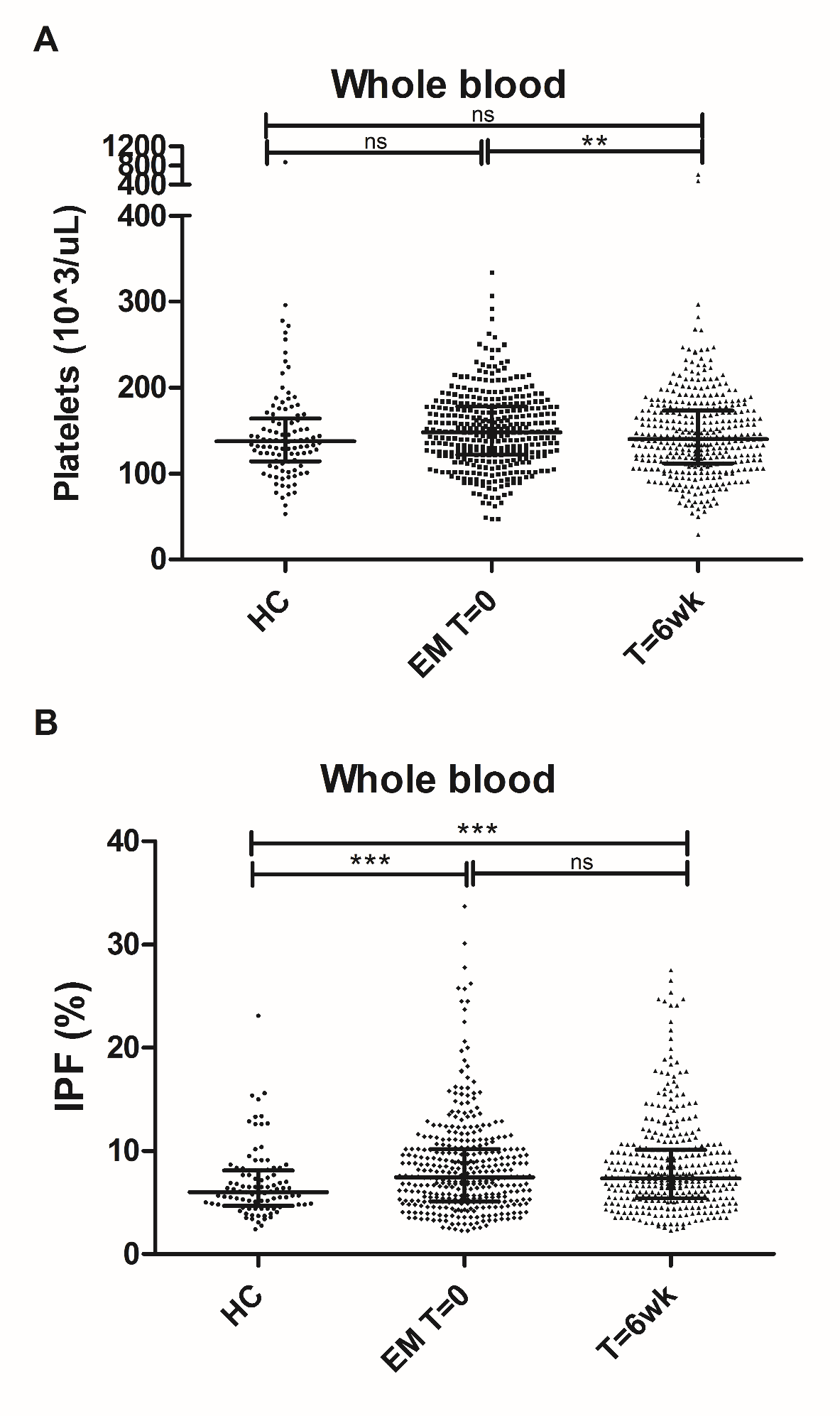


**Figure S6**, related to Fig 4
